# Case Report: A case of first-line treatment for rare ROS1 fusion mutation lung adenocarcinoma with entrectinib

**DOI:** 10.3389/fonc.2025.1566687

**Published:** 2025-04-16

**Authors:** Yifan Yang, Qing Yi, Wei Yang, Yongzhong Luo, Lemeng Zhang

**Affiliations:** ^1^ Thoracic Medicine Department 1, The Affiliated Cancer Hospital of Xiangya School of Medicine, Central South University/Hunan Cancer Hospital, Changsha, Hunan, China; ^2^ University of South China, Hengyang Medical School, Hengyang, Hunan, China; ^3^ Gene-seeq Research Institute, Nanjing Geneseeq Technology Inc, Nanjing, Jiangsu, China

**Keywords:** ROS1 fusion, entrectinib, lung adenocarcinoma, fusion mutation, intracranial lesions

## Abstract

The incidence of the ROS1 fusion mutation (ROS proto-oncogene 1) in non-small cell lung cancer (NSCLC) is approximately 1-2%. At least 55 partner genes have been identified that can fuse with ROS1.Herein, we report a case of a lung adenocarcinoma patient harboring a rare ROS1 fusion mutation with brain metastasis, who achieved good control of both lung and intracranial lesions after treatment with Entrectinib. A 68-year-old female patient with no smoking history presented with a cough and headache. She was diagnosed with advanced lung adenocarcinoma, stage T1cN3M1c IVb, which included multiple brain metastases. An IGR (downstream MAN1A1) ROS1:exon34 fusion was detected at a mutant allele frequency (MAF) of 12.73%, accompanied by two TP53 mutations c.1024C>T (p.R342*) and c.686_687del (p.C229Yfs*10). The resultant fusion protein preserves the whole TRKA kinase domain of ROS1, and therefore may constitutively activate ROS1.Therefore, RNA-seq was conducted to further confirm the expression of IGR-ROS1 fusion at mRNA level. A CD74:exon6 ~ ROS1: exon 35 fusion was identified, which could mediate the full kinase function. A biopsy of the right supraclavicular lymph node confirmed the diagnosis of lung adenocarcinoma. She was diagnosed with advanced lung adenocarcinoma, stage T1cN3M1c IVb, which included multiple brain metastases. The patient began treatment with entrectinib (600 mg, once daily) as a first-line therapy. At the time of diagnosis, the patient reported headaches and experienced sleep disturbances. Subsequently, the patient underwent whole-brain radiotherapy. Significant improvements were noted in her headache and insomnia symptoms. After one month, the longest diameter of the left upper lung nodule decreased from 19 mm to 12 mm, and there was a notable reduction in the right hilar and mediastinal lymph nodes. Additionally, the patient’s intracranial metastatic lesion reduced in size from 19 mm to 8 mm, leading to an improvement in her headache symptoms. It is worth further exploring whether patients carrying IGR fusions can receive targeted therapy.

## Introduction

The incidence of the ROS1 fusion mutation (ROS proto-oncogene 1) in non-small cell lung cancer (NSCLC) is approximately 1-2%. At least 55 partner genes have been identified that can fuse with ROS1. To date, at least 14 common ROS1 fusion partner genes have been recognized, including CD74 (44%), EZR (16%), SDC4 (14%), and SLC34A2 (10%) (1, 2). The ROS1 breakpoint is mostly conserved, with frequent fusion sites located in introns 31, 32, and 33. In contrast, rare partner gene fusions primarily occur in introns 34 and 35. Herein, we report a case of a lung adenocarcinoma patient harboring a rare ROS1 fusion mutation with brain metastasis, who achieved good control of both lung and intracranial lesions after treatment with Entrectinib.

## Case description

**Figure 1:**
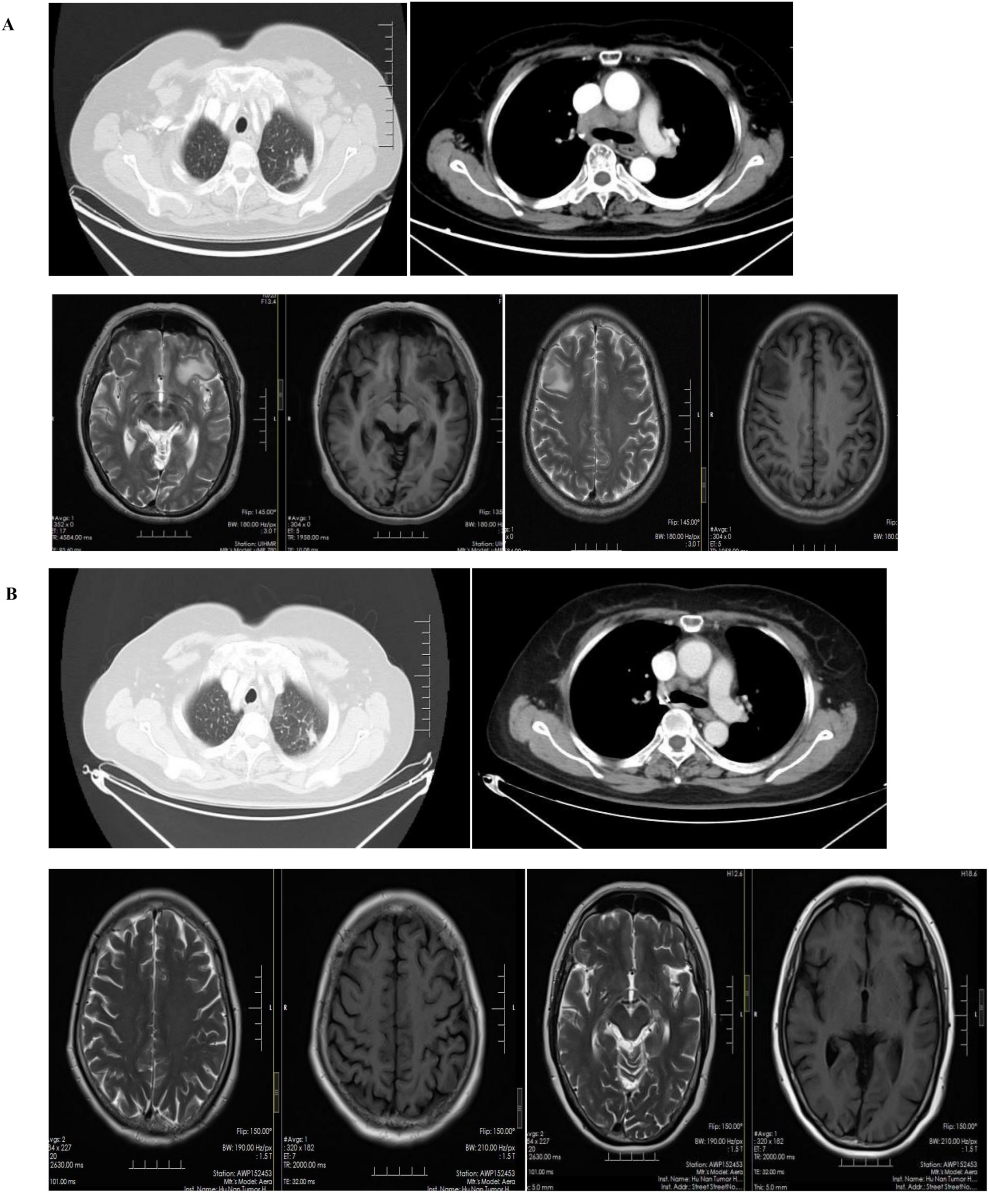
Radiographic alterations in the patient. **(A)**: Multiple brain metastases, pulmonary lesions and mediastinal lymphadenopathy before treatment. **(B)**: comparison of lung and intracranial lesions after treatment.

**Figure 2:**
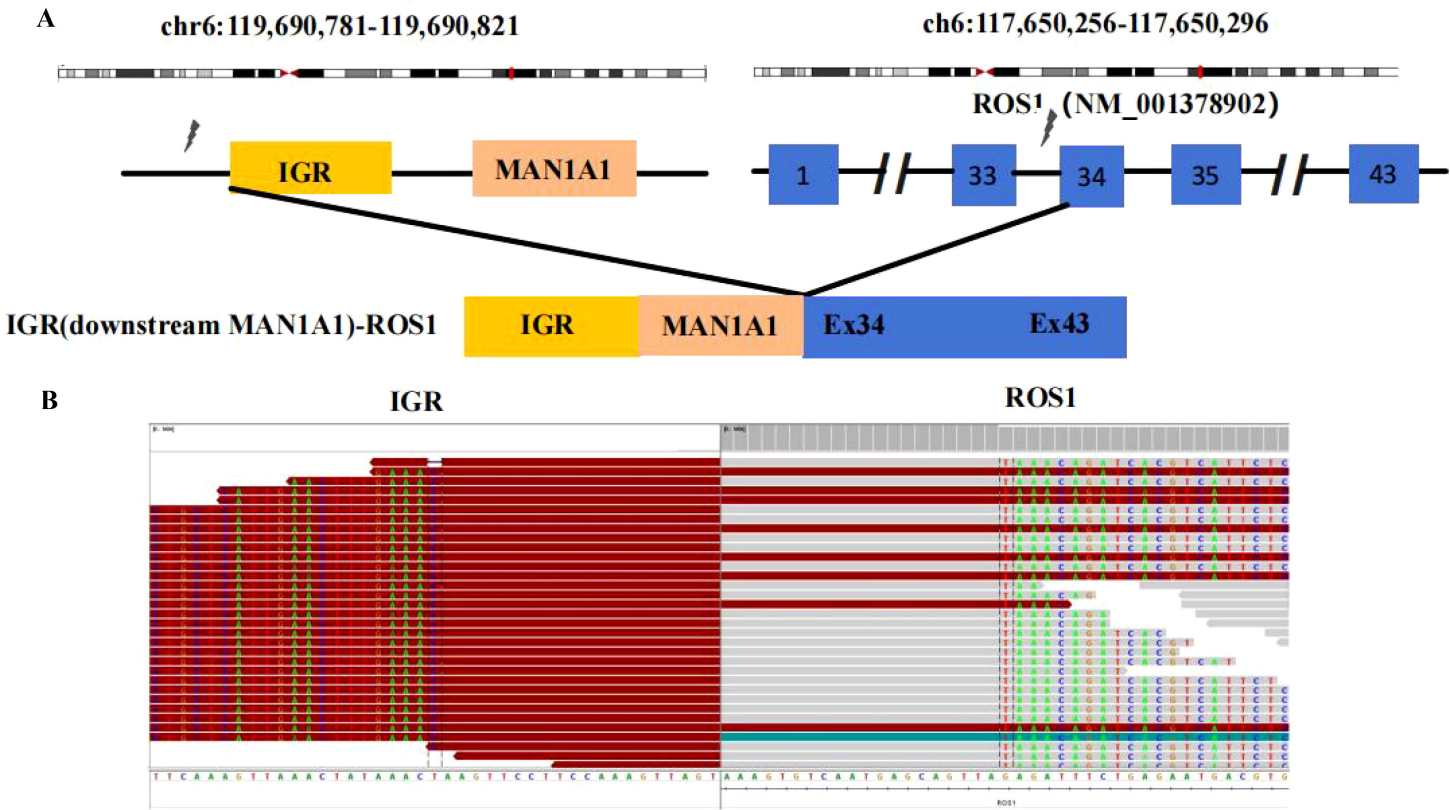
ROS1 fusion identified by DNA-based NGS. **(A)** Schematic representation of the IGR (downstream MAN1A1)-ROS1 fusion structure; **(B)** Sequencing reads of the IGR (downstream MAN1A1) and ROS1 are shown by the Integrative Genomics Viewer.

**Figure 3:**
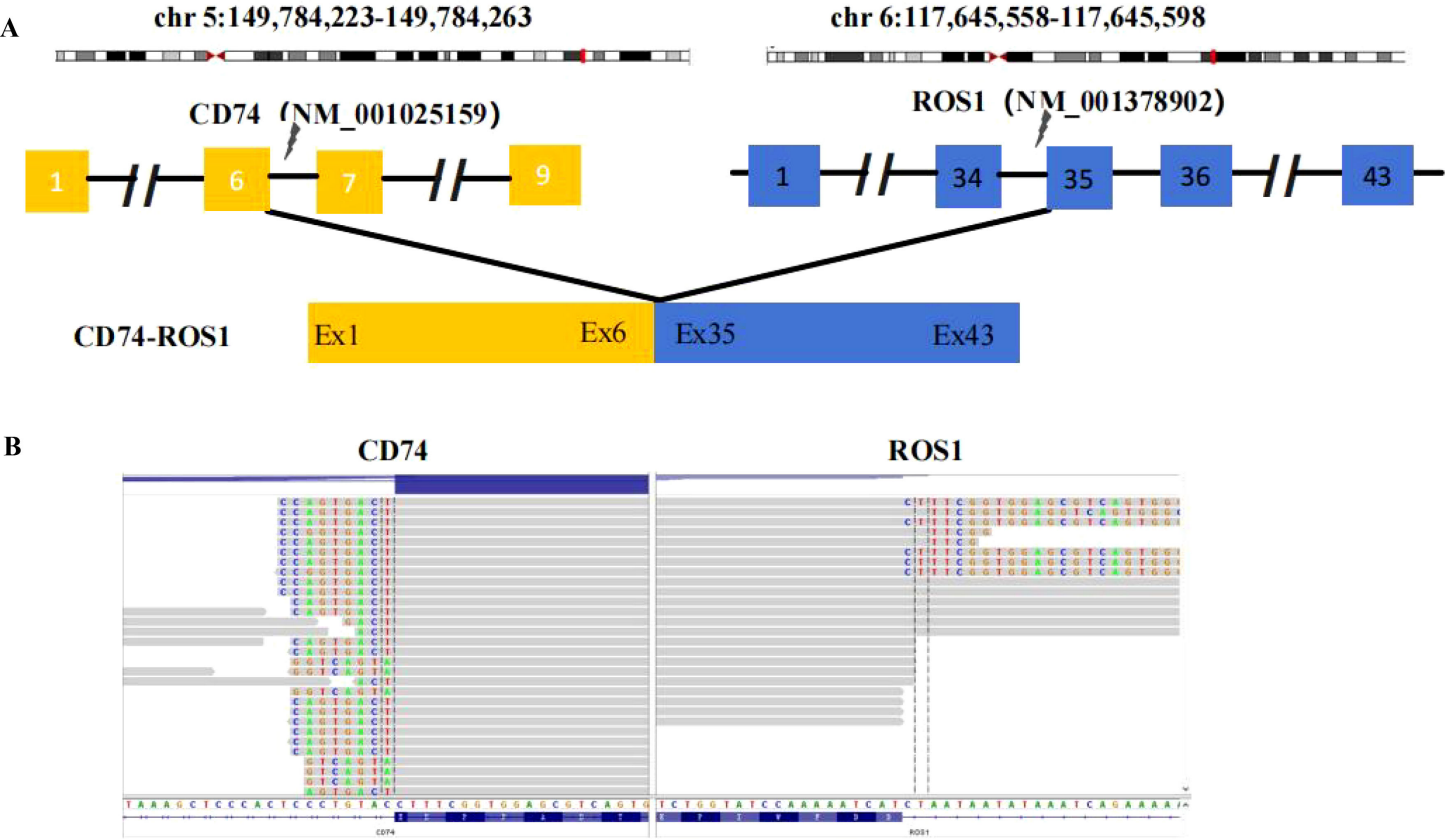
ROS1 fusion identified by RNA-based NGS. **(A)** Schematic representation of the CD74-ROS1 fusion structure. **(B)** Sequencing reads of the CD74 and ROS1 are shown by the Integrative Genomics Viewer.

A 68-year-old female patient with no smoking history presented with a cough and headache in May 2024. A biopsy of the right supraclavicular lymph node confirmed the diagnosis of lung adenocarcinoma. She was diagnosed with advanced lung adenocarcinoma, stage T1cN3M1c IVb, which included multiple brain metastases. The initial imaging of the lungs and brain is presented in **Figure 1A**. At the time of diagnosis, the patient reported headaches and experienced sleep disturbances. Subsequently, the patient underwent whole-brain radiotherapy. Based target capture of clinical NGS for 55 cancer-relevant genes (GeneseeqPrime). An IGR (downstream MAN1A1) ROS1:exon34 fusion (**Figure 2**) was detected at a mutant allele frequency (MAF) of 12.73%, accompanied by two TP53 mutations c.1024C>T (p.R342*) and c.686_687del (p.C229Yfs*10) (**Table 1**, **Figure 2**). The resultant fusion protein preserves the whole TRKA kinase domain of ROS1, and therefore may constitutively activate ROS1.Therefore, RNA-seq was conducted to further confirm the expression of IGR-ROS1 fusion at mRNA level. A CD74:exon6 ~ ROS1: exon 35 fusion was identified (**Figure 3**), which could mediate the full kinase function.

**Table 1:** Genetic testing results.

Gene	Aberration	Variant	Abundance
TP53	p.C229Yfs*10 exon7 frameshift mutation	c.686_687del (p.C229Yfs*10)	9.5%
TP53	p.R342* exon10 nonsense mutation	c.1024C>T (p.R342*)	8.54%
ROS1	IGR (downstream MAN1A1)~ROS1 fusion	IGR (downstream MAN1A1)~ROS1:exon34	12.73%

The symbol “*” denotes a premature translation termination.

The patient began treatment with entrectinib (600 mg, once daily) as a first-line therapy. Significant improvements were noted in her headache and insomnia symptoms. After one month, the longest diameter of the left upper lung nodule decreased from 19 mm to 12 mm, and there was a notable reduction in the right hilar and mediastinal lymph nodes. Additionally, the patient’s intracranial metastatic lesion reduced in size from 19 mm to 8 mm, leading to an improvement in her headache symptoms. Overall, the patient achieved a partial response (PR) to the treatment (**Figure 1B**).

As of now, she has been on entrectinib for five months, and both the lung and brain lesions have consistently remained in a state of partial response. The main adverse reactions experienced include grade 2 blurred vision and oral mucositis, both of which improved with symptomatic treatment.

## Discussion

Retrospective NGS findings in 17,158 Chinese NSCLC patients identified 258 (1.5%) with ROS1 fusion. Primary fusion partners included CD74 (38%), EZR (13%), SDC4 (13%), and SLC34A2 (10%). About 10 cases (5.4%) were found in the intergenic region (IGR), with partners like FAM65B and GRIK2. Three patients received crizotinib, and two had durable responses (3). IGRs are short, non-coding DNA sequences between genes, distinct from introns. Abundant in eukaryotes, they comprise a significant part of the human genome once deemed non-functional. Research reveals that some IGR sequences can affect gene expression and regulate nearby genes (4). ROS1 fusions typically occur in introns 30-34, preserving the kinase domain in exons 36-41. A novel fusion, IGR (downstream MAN1A1) ROS1:exon34, is reported. MAN1A1, near ROS1 on chromosome 6q22, encodes a Golgi 1,2-mannosidase, a type II transmembrane protein in the glycosyl hydrolase family 47. *In vitro*, the MAN1A1~ROS1 fusion protein shows strong transforming ability, and crizotinib dose-dependently inhibits its growth (5). We hypothesize that the MAN1A1 IGR may affect ROS1 gene expression, preserving its kinase domain via cis-regulatory elements, potentially causing cellular oncogenesis. It also responds positively to ROS1 kinase inhibitors.

Clinical trials such as BFAST, STARTRK-1, and STARTRK-2 have shown that entrectinib is highly effective in patients with ROS1-fusion lung adenocarcinoma. The ORR after treatment is 81.5%, along with significant improvements in PFS and OS (6). Entrectinib shows significant antitumor activity and good tolerability, especially in patients with CNS metastases, compared to crizotinib (7–9). Moreover, its effectiveness in patients with measurable baseline CNS metastases is comparable to that in patients who have not received local brain radiotherapy or who received brain radiotherapy at least six months before treatment with entrectinib (10). At ESMO in 2024, an integrated analysis of STARTRK-2, STARTRK-1, and ALKA-372-001 was presented. This analysis revealed that first-line treatment with entrectinib in ROS1-fusion NSCLC patients achieved a systemic ORR of 68.2%. Additionally, the median PFS was 17.5 months, while the median OS was 52.3 months. For patients with baseline CNS metastases, the intracranial ORR was 66.7%. The median intracranial PFS was 14.7 months, the systemic median PFS was 11.9 months, and the median OS was 29.6 months (11). In our case, the patient showed a positive response to entrectinib, particularly in terms of relief from CNS symptoms. Entrectinib has a weak interaction with P-glycoprotein (P-gp), which facilitates its ability to penetrate the blood-brain barrier. This characteristic provides a significant advantage in the treatment of CNS diseases.

To the best of our knowledge, this is the first report of an MAN1A1 ROS1 in exon34 fusion. For patients with rare ROS1 fusions, entrectinib has shown effectiveness in treating both lung lesions and intracranial lesions. Considering whether patients with IGR fusions can be received targeted therapies or not is always confused for oncologists. Therefore, we further conducted validation for the specific fusion. FISH, IHC or RNA-based NGS were the most commonly methods to confirmed ROS1 fusion. Considering RNA-based NGS for fusion detection can be directly converted to protein information which was used in this study. CD74:exon6 ROS1 in exon 35 fusion was successfully tested at the RNA level. Another interesting thing is the fusions detected by RNA-NGS was not consistent with those detected by DNA-NGS in this case. DNA-NGS could not effectively predict true fusion splicing patterns on RNA transcriptome based on detected breakpoint information. Chromothripsis or alternative splicing of complex fusions at RNA level might influence the outcome of transcription (12). Therefore, RNA-NGS is used to verify the generation of fusion proteins, providing more precise guidance for clinical treatment.

## Data Availability

The original contributions presented in the study are included in the article/supplementary material. Further inquiries can be directed to the corresponding author.

## References

[1] Li N Chen Z Huang M Zhang D Hu M Jiao F. Detection of ROS1 gene fusions using next-generation sequencing for patients with Malignancy in China. Front Cell Dev Biol. (2022) 10:1035033. doi: 10.3389/fcell.2022.1035033 36589752 PMC9798300

[2] Drilon A Jenkins C Iyer S Schoenfeld A Keddy C Davare MA. ROS1-dependent cancers - biology, diagnostics and therapeutics. Nat Rev Clin Oncol. (2021) 18:35–55. doi: 10.1038/s41571-020-0408-9 32760015 PMC8830365

[3] Gao D Han Y Zhao Z Ou Q Tong X Zhao R. Abstract 741: Molecular and clinicopathological characteristics of Chinese non-small cell lung cancers with ROS1 gene fusions identified by next-generation sequencing. Cancer Res. (2020) 80:741–1. doi: 10.1158/1538-7445.AM2020-741

[4] Hertz MI Thompson SR. Mechanism of translation initiation by Dicistroviridae IGR IRESs. Virology. (2011) 411:355–61. doi: 10.1016/j.virol.2011.01.005 PMC3076094 21284991

[5] Panagopoulos I Heim S. Interstitial deletions generating fusion genes. Cancer Genomics Proteomics. (2021) 18:167–96. doi: 10.21873/cgp.20251 PMC8126330 33893073

[6] Drilon A Siena S Dziadziuszko R Barlesi F Krebs MG Shaw AT. Entrectinib in ROS1 fusion-positive non-small-cell lung cancer: integrated analysis of three phase 1-2 trials. Lancet Oncol. (2020) 21:261–70. doi: 10.1016/s1470-2045(19)30690-4 PMC7811790 31838015

[7] Yu ZQ Wang M Zhou W Mao MX Chen YY Li N. ROS1-positive non-small cell lung cancer (NSCLC): biology, diagnostics, therapeutics and resistance. J Drug Targeting. (2022) 30:845–57. doi: 10.1080/1061186x.2022.2085730 35658765

[8] Zhao X Zhang X Chen H Bao H Wu X Wang H. Mechanisms of resistance to tyrosine kinase inhibitors in ROS1 fusion-positive nonsmall cell lung cancer. Clin Chem. (2024) 70:629–41. doi: 10.1093/clinchem/hvae008 38416709

[9] Drilon A Chiu CH Fan Y Cho BC Lu S Ahn MJ. Long-term efficacy and safety of entrectinib in ROS1 fusion-positive NSCLC. JTO Clin Res Rep. (2022) 3:100332. doi: 10.1016/j.jtocrr.2022.100332 35663414 PMC9160474

[10] Fischer H Ullah M de la Cruz CC Hunsaker T Senn C Wirz T. Entrectinib, a TRK/ROS1 inhibitor with anti-CNS tumor activity: differentiation from other inhibitors in its class due to weak interaction with P-glycoprotein. Neuro-oncology. (2020) 22:819–29. doi: 10.1093/neuonc/noaa052 PMC7283026 32383735

[11] Fan Y Drilon A Chiu C-H Loong HH Siena S Krzakowski M. Brief report: updated efficacy and safety data from an integrated analysis of entrectinib in locally advanced/metastatic ROS1 fusion-positive non–small-cell lung cancer. Clin Lung Cancer. (2024) 25:e81–e86.e84. doi: 10.1016/j.cllc.2023.12.001 38245456 PMC11733145

[12] Li W Liu Y Li W Chen L Ying J. Intergenic breakpoints identified by DNA sequencing confound targetable kinase fusion detection in NSCLC. J Thorac oncology: Off Publ Int Assoc Study Lung Cancer. (2020) 15:1223–31. doi: 10.1016/j.jtho.2020.02.023 32151779

